# Anatomy of the Crowd4Discovery crowdfunding campaign

**DOI:** 10.1186/2193-1801-2-560

**Published:** 2013-10-24

**Authors:** Ethan O Perlstein

**Affiliations:** Perlstein Lab, Independent Scientist, San Francisco, CA USA

## Abstract

Crowdfunding allows the public to fund creative projects, including curiosity-driven scientific research. Last Fall, I was part of a team that raised $25,460 from an international coalition of “micropatrons” for an open, pharmacological research project called Crowd4Discovery. The goal of Crowd4Discovery is to determine the precise location of amphetamines inside mouse brain cells, and we are sharing the results of this project on the Internet as they trickle in. In this commentary, I will describe the genesis of Crowd4Discovery, our motivations for crowdfunding, an analysis of our fundraising data, and the nuts and bolts of running a crowdfunding campaign. Science crowdfunding is in its infancy but has already been successfully used by an array of scientists in academia and in the private sector as both a supplement and a substitute to grants. With traditional government sources of funding for basic scientific research contracting, an alternative model that couples fundraising and outreach – and in the process encourages more openness and accountability – may be increasingly attractive to researchers seeking to diversify their funding streams.

## Why crowdfunding?

Online fundraising for creative projects is called crowdfunding (Wheat et al. 2012; Kaplan 2013). The mechanics of offline fundraising also apply to crowdfunding, namely fine-tuning a message and then appealing to the general public, except prospective “crowdfunders” are anyone with an Internet connection and a credit card. Since 2009, the public has patronized an array of projects by artists, technologists, entrepreneurs and others on websites like Kickstarter, which has collected $500 M in donations to date, with a 44% success rate. However, scientists, in particular biomedical and pharmaceutical researchers, have been slow to embrace crowdfunding, because the average sums raised by crowdfunding are several orders of magnitude smaller than research grants awarded by government agencies like National Institutes of Health (NIH). Also, crowdfunding requires dynamic online presence and social media savvy, which many academic and industry scientists don’t have time or incentives to cultivate.

Prior to last year, I too dismissed crowdfunding as a viable funding source for my research. But in the Spring of 2012, with less than six months of funding left in my fellowship at Princeton University and my future career path uncertain, I decided to perform my first crowdfunding experiment. This experiment began with the selection of a topic whose importance could be understood by the public, and that fit my research trajectory. After the publication of my former Princeton lab’s paper on the accumulation of the antidepressant Zoloft in yeast cells (Chen et al. 2012), I emailed dozens of prospective collaborators in order to extend this research. Because the amount I could realistically raise by crowdfunding would be a fraction of a traditional grant, I needed a collaborator with an existing lab infrastructure.

Eventually, Professor David Sulzer of Columbia University Medical Center and I struck up a correspondence. The Sulzer lab studies the mechanism of action of amphetamines. Over the years, they observed multilamellar bodies in mouse brain cells treated with amphetamines (Larsen et al. 2002; Cubells et al. 1994), These multilamellar bodies resemble membranous structures in yeast cells treated with Zoloft. Sulzer and I wondered if amphetamines and antidepressants, which are both hydrophobic weak bases, might accumulate in multilamellar bodies. I proposed autoradiography as an experimental technique to test this hypothesis. Daniel Korostyshevsky, a technician in my former Princeton lab who mastered electron microscopy techniques required to investigate drug-membrane interactions, signed on as lead experimentalist. With a team in place, the amphetamines distribution project would proceed in the Sulzer lab.

## Launching a campaign

Next, we built a crowdfunding campaign from the ground up, a process that started with setting a fundraising goal. I crunched the numbers on my 2007–2012, $1 M budget at Princeton. My average monthly “burn rate” is $17,000, split between labor and equipment costs (Perlstein 2012a). The R03 is a non-renewable grant of up to $50,000 awarded by NIH for small research projects, and the closest analog from the traditional grant world to what we were proposing. Based on the requirements of our project, we set an ambitious $25,000 goal. The centerpiece of our campaign was a project video, which I commissioned from a professional videographer, a sound artist, and a composer. Earlier forays into crowdfunding by artists and technologists demonstrated that a short video not only increased the probability of successful funding, but also allowed project leaders to present in their own words a compelling, jargon-free narrative. I also penned introductory blog posts on my lab website in the weeks leading up to launch, which resulted in pledges from enthusiastic supporters (Perlstein 2012b). The final pre-launch preparation was creating rewards for donations ranging from $10 to $1,000. For example, a $25 donation entitled one to a 3D-printed plastic model of a methamphetamine molecule.

Starting on October 4, 2012, the Crowdsourcing Discovery campaign (later renamed Crowd4Discovery) formally debuted on the crowdfunding site RocketHub. That evening we hosted a launch party to screen the project video in a New York City venue, similar to the way political candidates inaugurate their campaigns with an in-person gathering designed to capture donations and contact information of prospective evangelists. We originally set the campaign length at 45 days, but it was later extended to 52 days (due to a suspension of campaign activities in the wake of Superstorm Sandy).

## Donors to dollars

**Figure 1:**
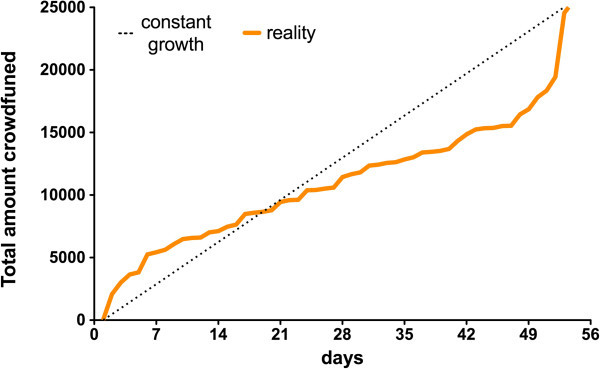
**Plot of daily fundraising totals over the duration of the Crowd4Discovery campaign.** The dashed line indicates idealized constant fundraising growth ($25,000/52 days).

Cumulatively, we raised $25,460 from 390 donors in 15 countries. The average donation is $64 and the median donation is $25. Figure 1 is a plot of daily fundraising totals over the campaign. As is characteristic of crowdfunding campaigns, half of the donations arrived in the beginning and end of the campaign. In fact, three phases are apparent in our fundraising trajectory. First, an opening burst during the first week netted us a 20% “down payment” that established our viability. Second, slow and steady linear growth persisted until the last week. Third, with 24 hours left and over $5,000 from our goal, we experienced a hockey-stick surge, with 130 donors propelling us over the finish line.

In keeping with general crowdfunding statistics, 93% of C4D supporters contributed less than $200. In fact, 63% of the 390 donors gave in the $5-$49 range. However, these small donors only amounted to 21% of the goal. So how did we make up the rest? 30% of donors contributed in the $50-$200 range, and they accounted for 44% of the goal. 7% of donors who contributed $200 or more accounted for 35% of the goal. As I’ll elaborate below, I estimate that 60% of all donors are in my social networks, namely Facebook and Twitter, while the remaining 40% are more than one degree removed – in other words, strangers.

Exact quantification is not possible post hoc, but according to an informal survey conducted in collaboration with National Public Radio (NPR), I learned more about our donor profile (Perlstein 2013a). 65% are men based on first-name analysis. 80% of respondents are age 25–44. Over 75% are from the anglophone countries USA, Canada, the UK, Australia and New Zealand. 52% of respondents self-identified as scientists, researchers or academic trainees. The next largest blocs were in computing and Internet technology, and management. The majority of respondents said they supported the C4D campaign because of a general desire to see alternative funding for scientific research.

## Marketing 101

News, online and social media played an essential role in attracting donors. I cold called (by email) science writers and journalists who had previously written about psychopharmacology or science crowdfunding. Persistence paid off, as the C4D campaign was featured in Wired, Scientific American, The Economist and Forbes. We were also the beneficiaries of organic buzz on science blogs and social commenting sites like Hacker News. For example, *In the Pipeline*, the well-trafficked blog by pharmaceutical scientist Derek Lowe, mentioned our campaign twice. Judging by the total number of times our project video was viewed, our donation page on RocketHub was visited over 50,000 times.

Complementing the external buzz, I also made targeted appeals to my social networks – family, friends and followers. For example, when I posted links to press pieces about our project, I made a note of who “liked” or retweeted the news item, and then followed up by email. Our campaign would not have been successful without vigorous turnout from my social networks (Perlstein 2013b). In my case, 17% of my Facebook friends donated, and 10% of my Twitter followers donated. Daniel and I also pounded the pavement in person. For example, I was a panelist at a science communication meeting that took place in London during the second month of our campaign. Incidentally, all of this campaign activity expanded my Twitter following and referred web traffic to my lab website, creating a virtuous cycle of community building and fundraising potential.

## Diversify your funding portfolio

Experience suggests that many scientists would consider the amount of work involved in creating and promoting a crowdfunding campaign to be daunting. In my case, I devoted several hours per day on our campaign; now that the project is underway, we have committed ourselves to public engagement, which also takes time. But I should emphasize that crowdfunding couples fundraising and outreach in a sustainable way, which is not the case for grants proposals that are reviewed by a few select peers behind closed doors. For those scientists who already engage in online activities like blogging and tweeting about their research, crowdfunding may be an attractive option given the bleak traditional funding outlook, especially so for younger scientists (Bourne 2013). Over time, crowdfunding will gradually emerge as one pillar in a more diversified funding portfolio. Put another way: today, 99% of scientists are 0% crowdfunded. It’s not possible to predict the carrying capacity of science crowdfunding, but I can predict that the combination of crowdfunding and outreach will benefit both scientists and the public in the long run.
